# Association of High Density Lipoprotein with Platelet to Lymphocyte and Neutrophil to Lymphocyte Ratios in Coronary Artery Disease Patients

**DOI:** 10.1155/2014/686791

**Published:** 2014-11-16

**Authors:** Jayesh H. Prajapati, Sibasis Sahoo, Tushar Nikam, Komal H. Shah, Bhumika Maheriya, Meena Parmar

**Affiliations:** ^1^Department of Cardiology, U. N. Mehta Institute of Cardiology and Research Centre, Civil Hospital Campus, Ahmedabad, Gujarat 380016, India; ^2^Department of Research, U. N. Mehta Institute of Cardiology and Research Centre, Civil Hospital Campus, Ahmedabad, Gujarat 380016, India

## Abstract

*Background*. We aimed to evaluate a relationship between platelet-lymphocyte ratio (PLR) and neutrophil-lymphocyte ratio (NLR) with high density lipoprotein (HDL) cholesterol levels in coronary artery disease (CAD) patients. *Methods*. A total of 354 patients with angiographically confirmed coronary blockages were enrolled in the study. Hematological indices and lipid profiling data of all the patients were collected. *Results*. We have observed significant association between HDL and PLR (*P* = 0.008) and NLR (*P* = 0.009); however no significant relationship was obtained with HDL and isolated platelet (*P* = 0.488), neutrophil (*P* = 0.407), and lymphocyte (*P* = 0.952) counts in CAD patients. The association was subjected to gender specific variation as in males PLR (*P* = 0.024) and NLR (*P* = 0.03) were highly elevated in low HDL patients, whereas in females the elevation could not reach the statistically significant level. The PLR (217.47 versus 190.3; *P* = 0.01) and NLR (6.33 versus 5.10; *P* = 0.01) were significantly higher among the patients with acute coronary syndrome. In young patients the PLR (*P* = 0.007) and NLR (*P* = 0.001) were inversely associated with HDL, whereas in older population only NLR (*P* = 0.05) had showed a significant association. *Conclusion*. We conclude that PLR and NLR are significantly elevated in CAD patients having low HDL levels.

## 1. Introduction

The pathophysiology of atheroma, a process causing hardening of arterial innermost layer, is triggered by the development of “fatty streaks” of cholesterol, fatty acids, and inflammatory and immune cells in the tunica intima. Clinically, atheroma could remain asymptomatic for decades and is difficult to diagnose until it reaches the highly advanced stage of arterial disease [1]. Coronary artery disease (CAD) occurs when the atheromatic lesion is ruptured and the released content starts occluding the vessel providing blood flow to myocardium [2].

Growing body of evidence has recognized circulating blood components such as white blood cell (WBC) subtypes (neutrophils, lymphocytes, eosinophils, and monocytes) and platelets as effective biomarkers of inflammatory processes involved in atherosclerosis [3]. These markers have furnished effective, simple, and relatively cheap tool for the diagnosis and prognosis of CAD events even in the asymptomatic individuals. Previous research had revealed significant association between coronary heart risk and platelet-lymphocyte ratio (PLR) and neutrophil-lymphocyte ratio (NLR) [4, 5].

High density lipoprotein (HDL)—one of the chief components of human lipoprotein class—is recently gaining ample scientific attention due to its antiatherogenic properties providing cardioprotection. The inverse relationship between HDL level and cardiovascular diseases is primary due to the phenomenon known as “reverse cholesterol transport” where the cholesterol efflux facilitated by apo A–I and HDL acceptors or by diffusion leads to its mobilization from cell membrane to hepatic storage [6]. HDL prevents oxidative modification of arterial wall low density lipoprotein (LDL) due to the presence of paraoxonase, glutathione-peroxidase, and apo A-1 [7]. In addition to this, HDL exerts antithrombotic activity by preventing platelet aggregation [8].

In spite of the vital role played by blood markers of inflammation and HDL in atherosclerosis none of the studies have documented the relationship between PLR, PWR, and NLR and HDL levels in patients with angiographically significant coronary artery stenosis.

## 2. Materials and Methods

This single center, observational, randomized prospective study consisted of 354 consecutive patients admitted to the U. N. Mehta Institute of Cardiology and Research Center from February 2013 to April 2014 with diagnoses of CAD. The protocol was approved by the local ethics committee at our institute and informed consent was obtained from all patients. After detailed physical examination the demographic characteristics, diagnostic data, echocardiographic measurement, and angiographic measurement were collected by trained personnel. The confirmation of CAD was done by coronary angiography where one or more vessels were ≥50% stenotic. Left ventricular ejection fraction grades were defined as follows: normal (≥60), mild (45–59), moderate (30–40), and severe (<30).

Complete blood count and biochemical values were evaluated from blood samples obtained by antecubital vein puncture. Subjects were advised to fast at least for twelve hours before blood investigations. Total leucocyte count and its subtypes including neutrophil, lymphocyte, and monocyte and platelet count were analyzed using an automated blood cell counter. Lipid values like total cholesterol (TC), triglycerides (TG), total lipid (TL), low density lipoprotein cholesterol (LDL-C), high density lipoprotein cholesterol (HDL-C), and very low density lipoprotein (VLDL) were measured by International Federation of Clinical Chemistry (IFCC) approved enzymatic methods using commercially available kit on autoanalyzer (ARCHITECH PLUS ci4100, Germany). The cutoff value for low HDL-C was selected as <40 mg/dL in males and <50 mg/dL in females as described earlier [9].

### 2.1. Statistical Methods

All statistical studies were carried out using SPSS program version 20 (Chicago, IL, USA). The NLR was calculated from the differential count by simply dividing the neutrophil measurement by lymphocyte measurement. In the same manner PLR was calculated by dividing platelet count by lymphocyte count. Quantitative variables were expressed as the mean ± standard deviation and qualitative variables were expressed as percentage (%). A comparison of parametric values between two groups was performed using the independent sample *t*-test. Categorical variables were compared using the chi-square test. A nominal significance was taken as a two-tailed *P* value < 0.05.

## 3. Results

Demographic details of the study population are shown in Table 1. Overall there were 284 male (80.2%) and 70 female (19.8%) patients showing a collective mean age of 52.71 ± 13.35 years and 5.9% of the in-hospital mortality rate. Acute coronary syndrome (ACS) was found in 83.1% of the population, whereas 16.9% of the patients were suffering from chronic stable angina (CSA). The distribution of various blood components in patients with low HDL-C and with normal HDL-C is presented in Table 2. The mean HDL-C levels in groups I and II were 47.53 ± 7.12 and 30.95 ± 7.43 mg/dL, respectively. The prevalence of hyperlow/high density lipoproteinemia was higher in males (93.4%) as compared to females (6.6%). We have observed that except for total WBC (per cmm) (12.35 ± 6.86 versus 11.35 ± 4.18; *P* = 0.033), PLR (201.6 ± 185.25 versus 177.77 ± 129.88; *P* = 0.008), and NLR (5.86 ± 5.02 versus 4.89 ± 3.64; *P* = 0.009) which were highly associated with low HDL-C none of the blood cells showed significant relationship with HDL-C in patients suffering from coronary stenosis. Moreover, no correlation was obtained between LV function and HDL-C levels.

The categorization of CSA and ACS patients according to the level of HDL-C is presented in Table 3. The total WBC counts, PLR, and NLR were significantly elevated in low HDL-C group in patients with ACS as indicated in Table 3. The mean total WBC, NLR, and PLR found in ACS patients having low HDL-C were 13.02 (per cmm), 6.33, and 217.47, respectively, significantly higher (total WBC, 11.81, NLR, 5.10, and PLR, 190.3) as compared to patients with normal HDL-C. The ACS patients having low HDL-C were more male (93.8%). However none of the isolated leukocyte subtypes and ratios showed noticeable correlation with HDL-C levels in CSA patients. We have observed that the association of HDL-C with circulating blood cell ratios is subjected to gender variation and is presented in Table 4. In males the HDL-C level showed significant inverse relationship between total WBC count (12.55 ± 6.9 versus 11.64 ± 4.2 per cmm; *P* = 0.01), PLR (206.06 ± 188.4 versus 184.9 ± 136.2; *P* = 0.024), and NLR (5.96 ± 5.1 versus 5.17 ± 3.82; *P* = 0.030), which was absent in case of female population. In the young group, the patients having low HDL-C were all male (100%) showing significant association with total WBC count (16.66 ± 11.97 versus 12.59 ± 4.91; *P* = 0.001), PLR (303.9 ± 322.2 versus 223.65 ± 181.6; *P* = 0.007), and NLR (8.37 ± 9.1 versus 5.1 ± 3.6; *P* = 0.001), whereas in case of older population none of the blood components showed a significant relation with HDL-C. In older patients more males (92.1%) were suffering from low HDL-C level as compared with females (7.9%) (Table 5).

## 4. Discussion

The presence of inflammatory and prothrombotic markers such as subset of WBC and platelets in circulation have proven their role as positive predictors of cardiovascular diseases as revealed by several epidemiological studies and various combinations of these markers often add higher degree of specificity to the predictive precision [10, 11]. The key finding of current investigation is the important association between increasing PLR and NLR with decreasing levels of HDL-C in CAD patients.

Population based and biological evidences of HDL-C being an independent and powerful negative predictor of CAD events are well documented and strong [12, 13]. The atheroprotective effect of HDL-C could be explained by the fact that inflammatory markers such as interleukin 6 and high sensitivity C-reactive protein are elevated in individuals having low HDL-C level [14]. The common risk factors of cardiovascular diseases such as poor dietary habits, lack of exercise, obesity, and tobacco and alcohol consumption are inversely involved with HDL-C levels [15, 16]. Moreover pharmacological interventions improving HDL-C are found to improve the cardiac outcome of patients suffering from CAD indicating the key role of HDL-C in development of atherosclerotic plaques [17].

Independent relationship between neutrophil, lymphocyte, monocyte, platelet, and CAD events had been reported by many [18, 19]. A study conducted on healthy Japanese population showed high correlation between elevated counts of blood cells and incidence of CAD [20]. Vasculogenesis, a process involved in ischemic injury, initiates various chronic adaptive processes such as elevation of circulating neutrophils which facilitates formation of aggregates between platelets and leucocytes in intravascular lumen in patients with acute coronary syndrome [21]. Platelets play pro-vital role in the process of atherothrombosis, atherosclerosis, and inflammation and are closely associated with vascular health. Activated platelets release proangiogenic mediators causing modulation of microvascular structure of blood vessels [22]. Pathologically hyperfunctioning of platelets in CAD patients exerts unfavorable outcomes that are often prevented by antiplatelet therapy [23]. Along with neutrophil and platelets circulating lymphocytes also provides significant information regarding the stratification of high risk cardiac patients. Ommen et al. [24] have documented a decrease in total and relative number of circulating lymphocytes during acute myocardial infarction and advanced congestive heart failure; this could be due to increased cortisol production induced by physiological stress.

Hence, the ratios of neutrophils and platelets to lymphocytes are powerful markers of atherosclerotic disease with neutrophil and platelet indicating the systemic inflammatory status and severity of thrombosis, respectively, and lymphocyte showing homeostasis of fibroproliferative responses along with overall inflammation [25–27]. In spite of their clear role in coronary atherosclerosis very few studies have addressed the type of relationship between NLR, PLR, and HDL-C levels in CAD patients. A study conducted on healthy, asymptomatic, young males showed higher levels of NLR among the individuals with low HDL-C, which is in accordance with our study [28]. Though there is no previous reporting of PLR and HDL-C association, high PLR has also emerged as an independent predictor of long-term survival of patients having myocardial infarction [29]. Demirag and Bedir had tried evaluating the comparative preoperative prognostic role of PLR and NLR in patients undergoing major vascular surgery and its association with the survival of the patients [30]. They found that increased levels of NLR and PLR were directly correlated with mortality and inversely correlated with survival in the postoperative period and that diabetic patients were under a higher risk. We found that PLR and NLR association with HDL-C was more prevalent in younger as well as ACS patients as documented by others also [31, 32].

In conclusion, we have observed a significant relationship between PLR and NLR with low HDL-C level in ACS patients. Furthermore we have found that in males and young patients this association is stronger in comparison to their female and older counterparts showing a role of low HDL-C induced inflammation in this subset of population. Though the underlined pathophysiological mechanism of increased PLR and NLR in patients with low HDL-C is not clear, recommendations of diet and exercise improving HDL-C levels are strongly advocated in this group of patients.

## Figures and Tables

**Table 1:** Demographic characteristics of the study population.

Parameters	Mean ± SD/*N* (%) *N* = 354
Age (years)	52.71 ± 13.35
Gender	
Male	284 (80.2)
Female	70 (19.8)
Left ventricular dysfunction	
Absent	76 (21.5)
Mild	109 (30.8)
Moderate	88 (24.9)
Severe	81 (22.9)
Diagnosis	
CSA	60 (16.9)
ACS	294 (83.1)
Outcome	
Discharge	333 (94.1)
Expired	21 (5.9)
Hemoglobin (gm/dL)	12.63 ± 2.21
WBC (per cmm)	11.56 ± 4.89
Total neutrophil (%)	73.25 ± 11.95
Total lymphocyte (%)	21.38 ± 14.41
Platelets (per cmm)	2.82 ± 1.03
Monocyte (%)	3.05 ± 1.27
Eosinophils (%)	2.63 ± 1.38
NLR	5.10 ± 3.99
PLR	182.89 ± 143.61
TC (mg/dL)	157.78 ± 47.33
Triglycerides (mg/dL)	132.00 ± 73.64
HDL (mg/dL)	34.51 ± 10.02
LDL (mg/dL)	96.86 ± 39.01
VLDL (mg/dL)	26.42 ± 14.73
LDL/HDL	3.03 ± 1.68
TC/HDL	4.88 ± 2.16
Total lipids (mg/dL)	641.43 ± 336.85

CSA: chronic stable angina, ACS: acute coronary syndrome, WBC: white blood cell, NLR: neutrophil-lymphocyte ratio, PLR: platelet-lymphocyte ratio, TC: total cholesterol, HDL: high density lipoprotein, LDL: low density lipoprotein, and VLDL: very low density lipoprotein. Level of significance was accepted at *P* < 0.05.

**Table 2:** Demographic, clinical, and laboratory characteristics of the population according to HDL-C levels.

Parameters	High density cholesterol (HDL-C)		*P* value
	Low HDL-C (*N* = 76)	Normal HDL-C (*N* = 278)	
	Mean ± SD/*N* (%)	Mean ± SD/*N* (%)	Significance
Age (years)	54.69 ± 14.29	52.17 ± 13.06	0.145
Gender			
Male	71 (93.4)	213 (76.6)	0.002
Female	5 (6.6)	65 (23.4)	
Left ventricular dysfunction			
Absent	14 (18.4)	62 (22.3)	0.56
Mild	25 (32.9)	84 (30.2)	0.75
Moderate	21 (27.6)	67 (24.1)	0.63
Severe	16 (21.1)	65 (23.4)	0.78
Diagnosis			
Chronic stable angina (CSA)	12 (15.8)	48 (17.3)	0.89
Acute coronary syndrome (ACS)	64 (84.2)	230 (82.7)	
Outcome			
Discharge	70 (92.1)	263 (94.6)	0.58
Expired	6 (7.9)	15 (5.4)	
Hemoglobin (gm/dL)	12.99 ± 2.55	12.54 ± 2.10	0.255
WBC (per cmm)	12.35 ± 6.86	11.35 ± 4.18	0.033
Neutrophil (%)	74.52 ± 12.70	72.91 ± 11.73	0.407
Lymphocyte (%)	20.06 ± 11.42	21.75 ± 15.12	0.952
Platelets (per cmm)	2.69 ± 1.0	2.86 ± 1.04	0.488
Monocyte (%)	2.93 ± 1.34	3.09 ± 1.25	0.411
Eosinophils (%)	2.51 ± 1.48	2.66 ± 1.369	0.900
NLR	5.86 ± 5.02	4.89 ± 3.64	0.009
PLR	201.60 ± 185.25	177.77 ± 129.88	0.008
HDL (mg/dL)	47.53 ± 7.12	30.95 ± 7.43	<0.0001
TC (mg/dL)	169.55 ± 48.89	154.56 ± 46.47	0.670
Triglycerides (mg/dL)	110.04 ± 57.99	138.00 ± 76.37	0.089
LDL (mg/dL)	100.36 ± 43.06	95.90 ± 37.86	0.086
VLDL (mg/dL)	22.00 ± 11.59	27.63 ± 15.27	0.086
LDL/HDL	2.15 ± 0.93	3.26 ± 1.76	0.52
TC/HDL	3.63 ± 1.04	5.22 ± 2.26	0.017
Total lipids (mg/dL)	626.74 ± 94.48	645.44 ± 376.98	0.385

CSA: chronic stable angina, ACS: acute coronary syndrome, WBC: white blood cell, NLR: neutrophil-lymphocyte ratio, PLR: platelet-lymphocyte ratio, TC: total cholesterol, HDL: high density lipoprotein, LDL: low density lipoprotein, and VLDL: very low density lipoprotein. Level of significance was accepted at *P* < 0.05.

**Table 3:** Demographic, clinical, and laboratory characteristics according to HDL-C levels in CSA versus ACS population.

Parameters	Chronic stable angina (CSA)			Acute coronary syndrome (ACS)		
	Low HDL (*N* = 12)	Normal HDL (*N* = 48)	*P* value	Low HDL (*N* = 64)	Normal HDL (*N* = 230)	*P* value
	Mean ± SD/*N* (%)	Mean ± SD/*N* (%)	Significance	Mean ± SD/*N* (%)	Mean ± SD/*N* (%)	Significance
Age (years)	55.08 ± 13.60	53.08 ± 13.01	0.77	54.62 ± 14.51	51.98 ± 13.09	0.14
Gender						
Male	11 (91.7)	35 (72.9)	0.57	60 (93.8)	178 (77.4)	0.003
Female	1 (8.3)	13 (27.1)		4 (6.3)	52 (22.6)	
Left ventricular dysfunction						
Absent	6 (50)	21 (43.8)	0.36	8 (12.5)	41 (17.8)	0.23
Mild	3 (25)	8 (16.7)	0.44	22 (34.4)	76 (33)	0.88
Moderate	0	5 (10.4)	0.03	21 (32.8)	62 (27)	0.08
Severe	3 (25)	14 (29.2)	0.02	13 (20.3)	51 (22.2)	0.69
Outcome						
Discharge	11 (91.7)	46 (95.8)	0.44	59 (92.2)	217 (94.3)	0.94
Expired	1 (8.3)	2 (4.2)		5 (7.8)	13 (5.7)	
Hemoglobin (gm/dL)	12.44 ± 3.77	11.93 ± 2.19	0.37	13.09 ± 2.28	12.66 ± 2.06	0.35
WBC (per cmm)	8.72 ± 3.20	9.16 ± 2.69	0.96	13.02 ± 7.16	11.81 ± 4.24	<0.01
Neutrophil (%)	68.91 ± 11.02	69.52 ± 11.33	0.92	75.57 ± 12.79	73.61 ± 11.72	0.50
Lymphocyte (%)	25.75 ± 9.94	24.47 ± 10.36	0.93	19.00 ± 11.44	21.18 ± 15.90	0.84
Platelets (per cmm)	2.43 ± 0.44	2.43 ± 0.87	1.00	2.74 ± 1.06	2.95 ± 1.06	0.92
Monocyte (%)	2.91 ± 1.08	3.12 ± 1.19	0.33	2.93 ± 1.40	3.08 ± 1.27	0.21
Eosinophils (%)	2.50 ± 1.00	2.87 ± 1.12	0.62	2.51 ± 1.56	2.62 ± 1.40	0.97
NLR	3.38 ± 2.18	3.84 ± 2.70	0.31	6.33 ± 5.26	5.10 ± 3.78	0.01
PLR	116.97 ± 72.72	117.75 ± 70.22	0.73	217.47 ± 195.7	190.30 ± 135.1	<0.01
TC (mg/dL)	153.09 ± 34.45	145.95 ± 42.66	0.45	172.64 ± 50.76	156.36 ± 47.11	0.59
Triglycerides (mg/dL)	118.44 ± 56.69	130.29 ± 76.86	0.658	108.47 ± 58.55	139.62 ± 76.33	0.10
LDL (mg/dL)	82.23 ± 30.81	88.71 ± 32.49	0.82	103.75 ± 44.35	97.40 ± 38.78	0.13
VLDL (mg/dL)	23.68 ± 11.31	26.04 ± 15.37	0.65	21.69 ± 11.71	27.97 ± 15.27	0.10
LDL/HDL	1.79 ± 0.78	3.00 ± 1.17	0.14	2.22 ± 0.94	3.32 ± 1.86	0.07
TC/HDL	3.30 ± 0.91	4.92 ± 1.55	0.07	3.69 ± 1.06	5.28 ± 2.38	0.03
Total lipids (mg/dL)	618.70 ± 81.16	733.63 ± 876.5	0.39	628.25 ± 97.27	627.04 ± 110.5	0.43

WBC: white blood cell, NLR: neutrophil-lymphocyte ratio, PLR: platelet-lymphocyte ratio, TC: total cholesterol, HDL: high density lipoprotein, LDL: low density lipoprotein, and VLDL: very low density lipoprotein. Level of significance was accepted at *P* < 0.05.

**Table 4:** Demographic, clinical, and laboratory characteristics according to HDL-C levels in male versus female population.

Parameters	Males			Females		
	Low HDL (*N* = 71)	Normal HDL (*N* = 213)	*P* value	Low HDL (*N* = 5)	Normal HDL (*N* = 65)	*P* value
	Mean ± SD/*N* (%)	Mean ± SD/*N* (%)	Significance	Mean ± SD/*N* (%)	Mean ± SD/*N* (%)	Significance
Age (years)	53.98 ± 14.48	50.29 ± 12.60	0.047	64.80 ± 4.43	58.33 ± 12.70	0.05
Left ventricular dysfunction						
Absent	13 (18.3)	39 (18.3)	0.85	1 (20)	23 (35.4)	0.02
Mild	24 (33.8)	64 (30)	0.65	1 (20)	20 (30.8)	1.00
Moderate	19 (26.8)	57 (26.8)	0.87	2 (40)	10 (15.4)	0.42
Severe	15 (21.1)	53 (24.9)	0.63	1 (20)	12 (18.5)	0.60
Diagnosis						
CSA	11 (15.5)	35 (16.4)	1.00	1 (20)	13 (20)	0.56
ACS	60 (84.5)	178 (83.6)		4 (80)	52 (80)	
Outcome						
Discharge	65 (91.5)	203 (95.3)	0.37	5 (100)	60 (92.3)	0.79
Expired	6 (8.5)	10 (4.7)		0	5 (7.7)	
Hemoglobin (gm/dL)	13.27 ± 2.14	12.98 ± 1.99	0.48	8.98 ± 4.50	11.09 ± 1.79	<0.01
WBC (per cmm)	12.55 ± 6.90	11.64 ± 4.20	0.01	9.46 ± 6.03	10.41 ± 4.01	0.37
Neutrophil (%)	74.78 ± 12.7	73.97 ± 11.31	0.26	70.8 ± 13.47	69.41 ± 12.28	0.63
Lymphocyte (%)	19.80 ± 11.38	20.87 ± 15.98	0.99	23.80 ± 2.73	24.63 ± 11.53	0.59
Platelets (per cmm)	2.74 ± 1.00	2.83 ± 1.09	0.318	2.02 ± 0.73	2.96 ± 0.89	0.80
Monocyte (%)	2.92 ± 1.37	3.08 ± 1.36	0.303	3.0 ± 1.0	3.12 ± 1.25	0.43
Eosinophils (%)	2.52 ± 1.52	2.61 ± 1.25	0.711	2.40 ± 0.54	2.83 ± 1.65	0.53
NLR	5.96 ± 5.10	5.17 ± 3.82	0.030	4.53 ± 3.66	3.97 ± 2.81	0.21
PLR	206.06 ± 188.4	184.90 ± 136.2	0.024	138.29 ± 128.1	154.42 ± 103.9	0.30
TC (gm/dL)	171.08 ± 49.90	153.22 ± 47.62	0.587	147.80 ± 24.62	158.96 ± 42.52	0.21
Triglycerides (mg/dL)	109.40 ± 58.45	138.47 ± 78.20	0.065	119.19 ± 56.07	136.46 ± 70.58	0.95
LDL (mg/dL)	102.67 ± 43.42	95.73 ± 39.02	0.125	67.45 ± 17.94	96.45 ± 34.06	0.10
VLDL (mg/dL)	21.88 ± 11.69	27.74 ± 15.64	0.062	23.83 ± 11.21	27.30 ± 14.12	0.95
LDL/HDL	2.22 ± 0.92	3.38 ± 1.85	0.059	1.20 ± 0.35	2.90 ± 1.39	0.11
TC/HDL	3.70 ± 1.04	5.36 ± 2.37	0.028	2.64 ± 0.56	4.74 ± 1.79	0.18
Total lipids (mg/dL)	626.97 ± 96.26	649.95 ± 427.0	0.340	623.51 ± 72.10	630.68 ± 12.76	0.75

CSA: chronic stable angina, ACS: acute coronary syndrome, WBC: white blood cell, NLR: neutrophil-lymphocyte ratio, PLR: platelet-lymphocyte ratio, TC: total cholesterol, HDL: high density lipoprotein, LDL: low density lipoprotein, and VLDL: very low density lipoprotein. Level of significance was accepted at *P* < 0.05.

**Table 5:** Demographic, clinical, and laboratory characteristics according to HDL-C levels in young versus old population.

Parameters	≤40 years of age			>40 years of age		
	Low HDL (*N* = 13)	Normal HDL (*N* = 53)	*P* value	Low HDL (*N* = 63)	Normal HDL (*N* = 225)	*P* value
	Mean ± SD/*N* (%)	Mean ± SD/*N* (%)	Significance	Mean ± SD/*N* (%)	Mean ± SD/*N* (%)	Significance
Age	33.00 ± 4.20	33.79 ± 4.17	0.65	59.17 ± 11.16	56.50 ± 10.40	0.46
Gender						
Male	13 (100)	48 (90.6)	0.57	58 (92.1)	165 (73.3)	0.003
Female	0	5 (9.4)		5 (7.9)	60 (26.7)	
Left ventricular dysfunction						
Absent	4 (30.8)	8 (15.1)	0.36	10 (15.9)	54 (24)	0.23
Mild	6 (46.2)	16 (30.2)	0.44	19 (30.2)	68 (30.2)	0.88
Moderate	0	18 (34)	0.03	21 (33.3)	49 (21.8)	0.08
Severe	3 (23.1)	11 (20.8)	0.84	13 (20.6)	54 (24)	0.69
Diagnosis						
CSA	2 (15.4)	9 (17)	0.78	10 (15.9)	39 (17.3)	0.93
ACS	11 (84.6)	44 (83)		53 (84.1)	186 (82.7)	
Outcome						
Discharge	12 (92.3)	53 (100)	0.44	58 (92.1)	210 (93.3)	0.94
Expired	1 (7.7)	0		5 (7.9)	15 (6.7)	
Hemoglobin (gm./dL)	13.84 ± 2.27	13.06 ± 2.07	0.57	12.81 ± 2.59	12.41 ± 2.09	0.26
WBC (per cmm)	16.66 ± 11.97	12.59 ± 4.91	0.001	11.66 ± 4.94	11.06 ± 3.94	0.17
Neutrophil (%)	76.92 ± 12.07	74.43 ± 10.85	0.86	74.03 ± 12.86	72.55 ± 11.93	0.41
Lymphocyte (%)	18.00 ± 10.55	23.45 ± 26.81	0.56	20.49 ± 11.63	21.35 ± 10.73	0.40
Platelets (per cmm)	3.13 ± 1.26	3.37 ± 1.14	0.97	2.60 ± 0.92	2.74 ± 0.99	0.51
Monocyte (%)	3.30 ± 1.65	3.00 ± 1.20	0.07	2.85 ± 1.28	3.11 ± 1.27	0.98
Eosinophils (%)	2.07 ± 0.64	2.50 ± 1.40	0.08	2.60 ± 1.29	2.70 ± 1.33	0.50
NLR	8.37 ± 9.10	5.10 ± 3.60	0.001	2.60 ± 1.29	2.70 ± 1.33	0.50
PLR	303.90 ± 322.2	223.65 ± 181.6	0.007	180.49 ± 137.0	166.97 ± 112.1	0.06
Total cholesterol (mg/dL)	172.27 ± 45.04	166.03 ± 52.51	0.64	168.99 ± 49.96	151.86 ± 44.63	0.42
Triglycerides (mg/dL)	121.76 ± 84.75	158.55 ± 76.77	0.96	107.63 ± 51.41	133.16 ± 75.63	0.07
HDL (mg/dL)	46.01 ± 8.08	29.94 ± 7.70	0.56	47.85 ± 6.94	31.19 ± 7.36	0.85
LDL (mg/dL)	104.21 ± 34.70	104.38 ± 41.98	0.72	99.56 ± 44.79	93.90 ± 36.64	0.03
VLDL (mg/dL)	24.35 ± 16.95	31.71 ± 15.35	0.96	21.52 ± 10.28	26.67 ± 15.13	0.06
LDL/HDL	2.37 ± 0.72	3.63 ± 1.44	0.14	2.11 ± 0.96	3.18 ± 1.82	0.15
TC/HDL	3.91 ± 0.83	5.75 ± 1.79	0.08	3.57 ± 1.08	5.09 ± 2.35	0.06
Total lipids (mg/dL)	637.75 ± 121.1	654.53 ± 117.1	0.85	624.97 ± 89.02	643.40 ± 415.5	0.39

CSA: chronic stable angina, ACS: acute coronary syndrome, WBC: white blood cell, NLR: neutrophil-lymphocyte ratio, PLR: platelet-lymphocyte ratio, TC: total cholesterol, HDL: high density lipoprotein, LDL: low density lipoprotein, and VLDL: very low density lipoprotein. Level of significance was accepted at *P* < 0.05.

## References

[1] Bonow R. O., Mann D. L., Zipes D. P., Libby P. (2011). *Braunwald's Heart Disease: A Textbook of Cardiovascular Medicine. The Vascular Biology of Atherosclerosis*.

[2] Hansson G. K., Hamsten A., Goldman L., Schafer A. I. (2011). *Atherosclerosis, Thrombosis, and Vascular Biology*.

[3] Madjid M., Awan I., Willerson J. T., Casscells S. W. (2004). Leukocyte count and coronary heart disease: implications for risk assessment. *Journal of the American College of Cardiology*.

[4] Papa A., Emdin M., Passino C., Michelassi C., Battaglia D., Cocci F. (2008). Predictive value of elevated neutrophil-lymphocyte ratio on cardiac mortality in patients with stable coronary artery disease. *Clinica Chimica Acta*.

[5] Gary T., Pichler M., Belaj K. (2013). Platelet-to-lymphocyte ratio: a novel marker for critical limb ischemia in peripheral arterial occlusive disease patients. *PLoS ONE*.

[6] Tall A. R. (1998). An overview of reverse cholesterol transport. *European Heart Journal*.

[7] Paoletti R., Gotto A. M., Hajjar D. P. (2004). Inflammation in atherosclerosis and implications for therapy. *Circulation*.

[8] Mineo C., Deguchi H., Griffin J. H., Shaul P. W. (2006). Endothelial and antithrombotic actions of HDL. *Circulation Research*.

[9] Dhanaraj E., Bhansali A., Jaggi S., Dutta P., Jain S., Tiwari P., Ramarao P. (2009). Predictors of metabolic syndrome in Asian north Indians with newly detected type 2 diabetes. *Indian Journal of Medical Research*.

[10] Soylu K., Yuksel S., Gulel O. (2013). The relationship of coronary flow to neutrophil/lymphocyte ratio in patients undergoing primary percutaneous coronary intervention. *Journal of Thoracic Disease*.

[11] Horne B. D., Anderson J. L., John J. M., Weaver A., Bair T. L., Jensen K. R., Renlund D. G., Muhlestein J. B. (2005). Which white blood cell subtypes predict increased cardiovascular risk?. *Journal of the American College of Cardiology*.

[12] Frohlich J., Dobiasova M. (2003). Fractional Esterification Rate of Cholesterol and Ratio of Triglycerides to HDL-Cholesterol Are Powerful Predictors of Positive Findings on Coronary Angiography. *Clinical Chemistry*.

[13] Barter P. (2004). Is high-density lipoprotein the protector of the cardiovascular system?. *European Heart Journal*.

[14] Higashi Y., Matsuoka H., Umei H. (2010). Endothelial function in subjects with isolated low HDL cholesterol: role of nitric oxide and circulating progenitor cells. *American Journal of Physiology - Endocrinology and Metabolism*.

[15] Ho M., Garnett S. P., Baur L. A., Burrows T., Stewart L., Neve M., Collins C. (2013). Impact of dietary and exercise interventions onweight change andmetabolic outcomes in obese children and adolescents a systematic review and meta-analysis of randomized trials. *JAMA Pediatrics*.

[16] Mammas I. N., Bertsias G. K., Linardakis M., Tzanakis N. E., Labadarios D. N., Kafatos A. G. (2003). Cigarette smoking, alcohol consumption, and serum lipid profile among medical students in Greece. *European Journal of Public Health*.

[17] von Eckardstein A., Assmann G. (2000). Prevention of coronary heart disease by raising high-density lipoprotein cholesterol?. *Current Opinion in Lipidology*.

[18] Madjid M., Fatemi O. (2013). Components of the complete blood count as risk predictors for coronary heart disease: in-depth review and update. *Texas Heart Institute Journal*.

[19] Selcuk H., Dinc L., Selcuk M. T., Maden O., Temizhan A. (2012). The relation between differential leukocyte count, neutrophil to lymphocyte ratio and the presence and severity of coronary artery disease. *Open Journal of Internal Medicine*.

[20] Prentice R., Szatrowski T. P., Fujikura T., Kato H., Mason M. W., Hamilton H. H. (1982). Leukocyte counts and coronary heart disease in a Japanese cohort. *American Journal of Epidemiology*.

[21] Siminiak T., Flores N. A., Sheridan D. J. (1995). Neutrophil interactions with endothelium and platelets: possible role in the development of cardiovascular injury. *European Heart Journal*.

[22] Kaplan Z. S., Jackson S. P. (2011). The role of platelets in atherothrombosis. *The American Society of Hematology*.

[23] Clappers N., Brouwer M. A., Verbeugt F. W. A. (2007). Antiplatelet treatment for coronary heart disease. *Heart*.

[24] Ommen S. R., Gibbons R. J., Hodge D. O., Thomson S. P. (1997). Usefulness of the lymphocyte concentration as a prognostic marker in coronary artery disease. *American Journal of Cardiology*.

[25] Pillay J., Kamp V. M., van Hoffen E. (2012). A subset of neutrophils in human systemic inflammation inhibits T cell responses through Mac-1. *The Journal of Clinical Investigation*.

[26] Shechter M., Merz C. N. B., Paul-Labrador M. J., Kaul S. (2000). Blood glucose and platelet-dependent thrombosis in patients with coronary artery disease. *Journal of the American College of Cardiology*.

[27] Drew A. F., Tipping P. G. (1995). T helper cell infiltration and foam cell proliferation are early events in the development of atherosclerosis in cholesterol-fed rabbits. *Arteriosclerosis, Thrombosis, and Vascular Biology*.

[28] Tok D., Iscen S., Ozenc S. (2014). Neutrophil—lymphocyte ratio is associated with low high-density lipoprotein cholesterol in healthy young men. *SAGE Open Medicine*.

[29] Azab B., Shah N., Akerman M., McGinn J. T. (2012). Value of platelet/lymphocyte ratio as a predictor of all-cause mortality after non-ST-elevation myocardial infarction. *Journal of Thrombosis and Thrombolysis*.

[30] Demirag M. K., Bedir A. (2013). Evaluation of preoperative neutrophil-lymphocyte ratio and platelet-lymphocyte ratio in patients undergoing major vascular surgery. *Turkish Journal of Thoracic and Cardiovascular Surgery*.

[31] Zazula A. D., Précoma-Neto D., Gomes A. M., Kruklis H., Barbieri G. F., Forte R. Y., Langowiski A. R., Facin G., De Souza L. C. G., Neto J. R. F. (2008). An assessment of neutrophils/lymphocytes ratio in patients suspected of acute coronary syndrome. *Arquivos Brasileiros de Cardiologia*.

[32] Oylumlu M., Yıldız A., Oylumlu M. (2014). latelet to lymphocyte ratio is a predictor of in-hospital mortality patients with acute coronary syndrome. *Anadolu Kardiyoloji Dergisi*.

